# Pyroptosis-Related Gene Signatures and Immune Modulation in Ovarian Cancer: Insights from Multi-Omics and Machine Learning

**DOI:** 10.3390/genes17050595

**Published:** 2026-05-21

**Authors:** Rakesh Arya, Viplov Kumar Biswas, Hemlata Shakya, Jong-Joo Kim

**Affiliations:** 1 Department of Biotechnology, Yeungnam University, Gyeongsan 38541, Gyeongbuk, Republic of Korea; 2 Department of Pathology and Laboratory Medicine, Emory University School of Medicine, Whitehead Biomedical Research, 615 Michael Street, Atlanta, GA 30322, USA; viplov.kumar.biswas@emory.edu; 3 Department of Biomedical Engineering, Shri G. S. Institute of Technology and Science, Indore 452003, Madhya Pradesh, India; hemlata.shakya19@gmail.com

**Keywords:** ovarian cancer, pyroptosis, immune infiltration, machine learning, CEP55, biomarker, scRNA-seq

## Abstract

**Background:** Ovarian cancer (OVCA) remains the most lethal gynecologic malignancy, with poor prognosis largely due to late-stage diagnosis and therapy resistance. Pyroptosis, a pro-inflammatory form of programmed cell death, has recently emerged as a regulator of tumor progression and immune regulation. This study aimed to systematically profile pyroptosis-related genes and identify robust biomarkers for OVCA. **Methods:** Microarray data from the GSE54388 dataset were analyzed to characterize pyroptosis-related gene expression. Immune cell infiltration was assessed using xCell, and pathway enrichment was performed via Gene Set Enrichment Analysis (GSEA). Weighted Gene Co-expression Network Analysis (WGCNA) identified hub genes, followed by Gene Ontology (GO) and Reactome enrichment. Machine learning algorithms (Support Vector Machine, XGBoost, and Generalized Linear Model) were employed for feature selection and biomarker identification. Validation was conducted across independent bulk and scRNA-seq datasets, with GEPIA2 used to compare OVCA and normal samples and KMplot for survival analysis. **Results:** OVCA samples showed significantly reduced infiltration of CD4^+^ and CD8^+^ T cells, mast cells, monocytes, neutrophils, and immature dendritic cells compared to normal samples. GSEA revealed enrichment of cell cycle-related pathways, implicating pyroptosis-related genes as key regulators of mitotic progression. From 1097 differentially expressed genes, 22 pyroptosis-related DEGs (PYRDEGs) were identified, with nine hub genes (CASP1, CEP55, CHMP4C, HTRA1, IL18, MELK, PKM, PTX3, TNFSF13B) strongly associated with OVCA. Functional enrichment linked these genes to cytokinesis, inflammasome activity, and immune signaling. Machine learning consistently identified CEP55 as the core biomarker, demonstrating high diagnostic accuracy (AUC up to 0.972) and significant upregulation in OVCA samples. Correlation analysis linked CEP55 expression to altered immune cell populations, including positive associations with Th1 and class-switched memory B-cells and negative associations with iDCs, Tregs, and M2 macrophages. CEP55 was highly expressed across bulk and scRNA-seq datasets (cancer epithelial and CD8+ TEMRA cells) and negatively correlated with overall survival (OS) and progression-free survival (PFS). **Conclusions:** Pyroptosis-related genes play pivotal roles in OVCA pathogenesis. CEP55 emerges as a promising biomarker for early detection and a potential therapeutic target, bridging cell cycle regulation with immune modulation.

## 1. Introduction

Ovarian cancer (OVCA) is the deadliest malignancy of the female reproductive system and ranks as the eighth most common cancer among women worldwide [1]. Prognosis for High-Grade Serous Ovarian Cancer (HGSOC) remains especially challenging due to its aggressive nature, frequent late-stage diagnosis, and the emergence of drug resistance [2]. According to the GLOBOCAN database, 324,603 new cases and 206,956 deaths from OVCA were reported globally in 2022. By 2050, the number of women worldwide diagnosed with ovarian cancer is projected to increase by more than 55%, reaching 503,448 cases. Annual deaths from ovarian cancer are expected to rise to 350,956, nearly a 70% increase compared to 2022 [3]. Current treatments, including surgery and platinum-based chemotherapy, have not significantly improved five-year survival rates [4]. These limitations underscore the urgent need for early diagnostic and prognostic biomarkers to enhance clinical outcomes and enable the development of targeted therapies.

Pyroptosis, a recently characterized form of programmed cell death, involves activation of gasdermin (GSDM) family proteins and caspases, leading to membrane permeabilization, cell swelling, and the release of intracellular inflammatory cytokines such as IL-18, IL-1β, HMGB1, and ATP. These events trigger a strong inflammatory response and promote immune cell infiltration [5,6]. Alterations in pyroptosis-related genes have a significant impact on tumor progression and have been shown to influence prognostic outcomes across various cancers, including OVCA [7]. Pyroptosis occurs via a classical caspase-1-dependent pathway initiated by PRRs, and a non-classical pathway where caspase-4, -5, and -11 directly cleave GSDMD, leading to cell rupture and inflammation [8,9]. The key effector molecules such as inflammasomes [10], Gasdermin family proteins (GSDMA, GSDMB, GSDMC, GSDMD, GSDME, and DFNB59) [11], and pro-inflammatory cytokines facilitate this process, with GSDMC switching apoptosis to pyroptosis and promoting tumor necrosis [12]. In addition, danger signals released by tumor-infiltrating macrophages and dendritic cells can activate GSDMD-mediated pyroptosis in tumor-infiltrating lymphocytes (TILs), thereby enhancing antigen presentation and immune response [13,14,15]. Initially recognized as a host defense mechanism against infection, pyroptosis is increasingly understood to play a role in tumorigenesis, invasion, and metastasis through its core components: inflammasomes, gasdermin proteins, and pro-inflammatory cytokines [13].

The tumor microenvironment is pivotal in driving cancer progression and comprises a dynamic ecosystem of cancer cells, stromal cells (e.g., fibroblasts, endothelial cells, mesenchymal stem cells), immune and inflammatory cells (e.g., lymphocytes, macrophages, myeloid cells), extracellular matrix components, and secreted factors such as immune checkpoints (PD-1/L1, CTLA4), chemokines, cytokines, and growth factors [16]. Since pyroptosis is a complex process involving multiple steps and tightly coordinated gene interactions, earlier research was limited to identifying only a few biomarkers due to technical constraints. However, comprehensive reanalysis of publicly available datasets from the GEO database now enables deeper investigation into the interplay between pyroptosis and immune responses. Recognizing immune cell infiltration patterns mediated by pyroptosis is crucial for uncovering the underlying mechanisms of OVCA progression and updating immunotherapeutic strategies. Recent advances in microarray-based profiling and high-throughput sequencing technologies have enabled the identification of differentially expressed genes (DEGs) and uncovered key biological mechanisms, paving the way for discovering promising biomarkers for cancer diagnosis, treatment, and prognosis.

This study systematically analyzed the expression profiles of pyroptosis-related differentially expressed genes (PYRDEGs) in OVCA using advanced machine learning techniques to build predictive models and identify hub genes critical to disease progression. Gene expression data from human high-grade serous ovarian cancer (HGSOC) and normal ovarian surface epithelium (OSE) samples were used to identify pyroptosis related hub-genes and their biological functions were examined through GSEA, Gene Ontology (GO) and Reactome pathway enrichment. Hub genes were further identified using WGCNA. Three machine learning algorithms: Support Vector Machines (SVM), eXtreme Gradient Boosting (XGBoost), and Generalized Linear Models (GLM) consistently identified CEP55 as the core gene, which demonstrated strong diagnostic performance in receiver operating characteristic (ROC) curve analysis and was validated in independent bulk and single-cell RNA datasets. CEP55 expression was significantly elevated in OVCA samples and showed a negative correlation with overall survival (OS) and progression-free survival (PFS). These findings highlight CEP55 as a promising biomarker for early detection and a potential therapeutic target in OVCA.

## 2. Materials and Methods

### 2.1. Data Source and Normalization

**Figure 1:**
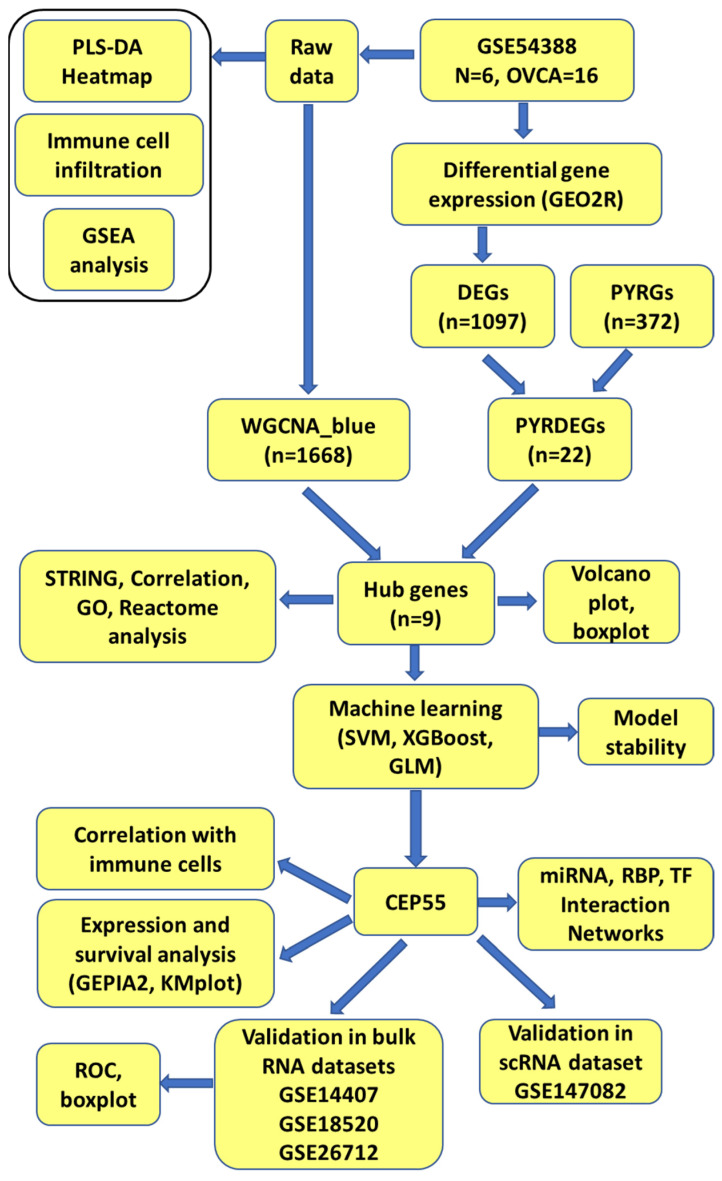
The schematic diagram illustrates the bioinformatics workflow used to identify and validate pyroptosis-related gene signatures in ovarian cancer.

Microarray data for the GSE54388 dataset, which includes six normal ovarian tissue samples and sixteen ovarian cancer (OVCA) patient tissue samples, were retrieved from the Gene Expression Omnibus (GEO) database (https://www.ncbi.nlm.nih.gov, accessed on 25 March 2025) [17] using R software (version 4.4.3). Heatmap and Partial Least Squares Discriminant Analysis (PLS-DA) plots were generated using the MetaboAnalyst web platform (version 6.0, https://www.metaboanalyst.ca, accessed on 3 June 2025) with sum normalization, log transformation, and autoscaling applied [18]. Three additional GEO datasets (GSE14407, GSE18520, and GSE26712) served as external validation cohorts. The GSE14407 dataset comprises twelve normal ovarian samples and twelve OVCA samples [19]; GSE18520 includes ten normal samples and fifty-three OVCA samples [20]; and GSE26712 contains ten normal and 185 OVCA samples [21]. The single-cell dataset GSE147082, which includes six samples, was used to analyze the distribution of CEP55 in ovarian cancer tissues. A schematic overview of the study’s methodological workflow is shown in Figure 1.

### 2.2. Immune Cell Infiltration Analysis

To further investigate the immune microenvironment between OVCA and normal samples, we analyzed immune cell infiltration in the GSE54388 dataset using xCell (https://comphealth.ucsf.edu/app/xcell, accessed on 16 June 2025) [22]. The results were visualized via boxplots, with statistical significance set at *p* < 0.05.

### 2.3. Gene Set Enrichment Analysis (GSEA)

To explore differences in signaling pathways, GSEA was performed using GSEA software version 4.4.0. Key parameters included a seed value of 2024, 1000 permutations, and gene sets containing between 15 and 500 genes. The Reactome subset “c2.cp.reactome.v2024.1.Hs.symbols.gmt” from the Molecular Signature Database (MsigDB), which contains 1787 annotated gene sets, was used for pathway analysis [23].

### 2.4. Identification of Differentially Expressed Genes (DEGs) and Pyroptosis-Related Genes (PYRGs)

Differential gene expression analysis was conducted using the GEO2R web tool (https://www.ncbi.nlm.nih.gov/geo/geo2r, accessed on 18 March 2025) [17] on the GSE54388 dataset. Probe sets without corresponding gene symbols were excluded. Genes with log_2_ fold change ≥±1 and *p* < 0.05 were classified as DEGs. Pyroptosis-related genes (*n* = 372) were obtained from Hei et al. (Table S1) [24]. The DEGs (*n* = 1097) were intersected with PYRGs using a Venn diagram to identify OVCA-associated pyroptosis-related DEGs (PYRDEGs).

### 2.5. Weighted Gene Co-Expression Network Analysis (WGCNA)

WGCNA was conducted using the “WGCNA” R package to identify gene modules associated with OVCA in the GSE54388 dataset. Expression and phenotype data were retrieved via the GEOquery package, followed by preprocessing to remove invalid gene symbol probes, average duplicate entries, exclude low-expression genes using goodSamplesGenes, and apply quantile normalization. A soft-thresholding power (β) was selected based on the scale-free topology criterion (signed R^2^ ≥ 0.8). The network was constructed using the blockwiseModules function with a minimum module size of 30, a merge cut height of 0.25, and an unsigned TOMType. Module eigengenes were calculated and correlated with clinical traits (normal and OVCA). Within the blue module, hub genes were filtered using thresholds of module membership (MM) > 0.6 and gene significance (GS) > 0.5 [25].

### 2.6. Identification and Analysis of Hub-Genes, Volcano Plot, Boxplot, Protein–Protein Interaction (PPI) Network, and Correlation

Hub genes were identified by intersecting the PYRDEGs (*n* = 22) with genes from the blue module (*n* = 1445) in the WGCNA. Results were visualized using volcano and boxplots. A PPI network was constructed via the STRING database (https://www.string-db.org, accessed on 30 April 2026), using a minimum required confidence score of 0.4 [26]. Correlation analysis was performed using SRplot (https://www.bioinformatics.com.cn, accessed on 30 April 2026), with statistical significance set at *p* < 0.05 [27].

### 2.7. Functional Enrichment Analysis of Hub-Genes

Functional enrichment analysis of hub genes was performed using the ShinyGO web tool (version 0.82, https://bioinformatics.sdstate.edu/go, accessed on 4 June 2025), including Gene Ontology (GO) terms for biological processes (BP), cellular components (CC), and molecular functions (MF), along with Reactome pathway analysis. Significance was determined based on fold enrichment and −log_10_ (FDR), with GO terms considered enriched at FDR < 0.05.

### 2.8. Identification of Core Pyroptosis-Related Hub-Gene by Machine Learning

Gene expression data from three OVCA cohorts (GSE14407, GSE18520, and GSE26712) were merged. Batch effects were corrected using ComBat, and values were standardized by z-scaling. The dataset was split into training (80%) and test (20%) sets with stratified sampling. Machine learning algorithms were developed in R using the caret framework with repeated five-fold cross-validation, ensuring feature selection occurred strictly within the CV loop. We used three machine learning algorithms: Support Vector Machines (SVM) [28], eXtreme Gradient Boosting (XGBoost) [29], and Generalized Linear Models (GLM) [30]. Model performance was assessed by ROC curves, boxplots of residuals, and reverse cumulative residual distributions. Core pyroptosis-related hub genes were identified by intersecting top-ranked features across all three algorithms.

### 2.9. Diagnostic Potential, Validation, and Immune Cell Correlation of CEP55

To evaluate the diagnostic potential of CEP55, analysis was conducted across three independent GEO datasets: GSE14407, GSE18520, and GSE26712 using the R package ‘pROC’ [31]. The ground truth labels were defined by clinical phenotype: Normal ovarian tissue was coded as the negative class, and OVCA tissue as the positive class. Validation was also performed in these datasets and boxplots depicting median and interquartile ranges were generated. Correlation between hub genes and immune cell types was analyzed using Spearman’s rank correlation [32].

### 2.10. Construction of mRNA-miRNA, mRNA-RBP, and mRNA-TF Interactions Networks

MiRNAs targeting CEP55 were predicted using the miRDB database (https://www.mirdb.org, accessed on 30 April 2026), with interactions retained for Target Scores ≥ 90 [33]. RNA-binding proteins (RBPs) interacting with CEP55 were predicted via the ENCORI database (https://www.rnasysu.com/encori, accessed on 2 June 2025), applying filters for clusterNum > 1 and clipExpNum > 1 [34]. Transcription factors (TFs) binding to CEP55 were identified using CHIPBase (version 3, https://rnasysu.com/chipbase3, accessed on 2 June 2025) [35] and hTFtarget (https://bioinfo.life.hust.edu.cn/hTFtarget, accessed on 2 June 2025) [36], with overlapping TFs were selected for further analysis. All networks were visualized in Cytoscape (version 3.10.4), and the top 2 influential nodes interacting with CEP55 were quantified using the CytoHubba plugin, based on degree, betweenness, closeness, and maximal clique centrality (MCC).

### 2.11. scRNA-Seq Data Processing and Analysis

For single-cell RNA sequencing (scRNA-seq) validation analysis, we employed the R package Seurat (version 5.4.0) in R [37]. The CreateSeuratObject() function was applied to construct the Seurat object from merged count matrices, while PercentageFeatureSet() was used to calculate the proportion of mitochondrial genes. The raw data had already been filtered by the uploader (≥600 genes per cell, ≤7.8% mitochondrial counts) [38]. Quality control was visualized using scatterplots of sequencing depth versus mitochondrial percentage and feature counts, as well as violin plots of nCount_RNA and nFeature_RNA. Normalization was performed with the LogNormalize method (NormalizeData()), and the top 1500 highly variable genes were identified using FindVariableFeatures(). These variable features were visualized with ggplot2, highlighting the top 10 genes. Dimensionality reduction was conducted using PCA (RunPCA()), with significant principal components determined by Dimheatmap, JackStrawPlot, and ElbowPlot. Cell clustering was performed with FindNeighbors() and FindClusters() (resolution = 0.5), followed by t-SNE (RunTSNE()) using the first 18 PCs, as determined by the elbow and JackStraw analyses. Cluster-specific marker genes were identified with FindAllMarkers() (adjusted *p* < 0.05, |log_2_FC| > 1, min.pct > 0.25). Heatmaps of the top 10 marker genes per cluster were generated with DoHeatmap(). Cell type annotation was carried out using SingleR (version 2.12.0) [39] with the HumanPrimaryCellAtlasData() reference (14 out of 37 main cell types) from celldex, combined with manual annotation based on literature-derived marker gene panels [40] for 14 ovarian cancer-relevant cell types (Mesenchymal cells, Fibroblasts, Epithelial cells (cancer cell), Epithelial cells (cancer stem), T cell: CD8+ effector memory RA, T cell: gamma-delta, Monocyte: leukotriene D4, B cell: Naïve, B cell: Plasma cell, Endothelial cells (lymphatic), Neuroepithelial cell: ESC-derived, Neurons: ES cell-derived neural precursor, MSC, Tissue stem cells: BM MSC). Finally, gene expression patterns of interest were visualized using DotPlot() and FeaturePlot().

### 2.12. Gene Expression and Survival Analysis

To evaluate gene expression and survival analysis, the GEPIA2 database (http://gepia2.cancer-pku.cn, accessed on 27 March 2026 [41] and the Kaplan–Meier Plotter database (https://kmplot.com/analysis, accessed on 27 March 2026) [42] were used.

### 2.13. Statistical Analysis

All statistical analyses were performed using R software (version 4.4.3). Group comparisons were conducted using the Wilcoxon rank-sum test, with *p* < 0.05 considered significant. In boxplots and heatmaps, asterisks were used to denote significance levels: **** (*p* < 0.0001), *** (*p* < 0.001), ** (*p* < 0.01), * (*p* < 0.05), and ns (*p* > 0.05).

## 3. Results

### 3.1. Raw Data Analysis and Clustering

**Figure 2:**
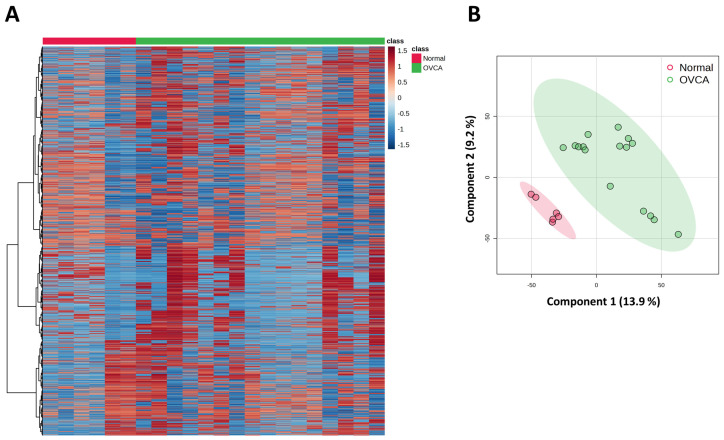
Gene expression-based clustering and classification of normal and OVCA samples. (**A**) Heatmap of differentially expressed genes across all samples, where red indicates upregulated expression and blue indicates downregulated expression. (**B**) PLS-DA scatter plot shows separation between OVCA (green circles) and normal (red circles) samples along Component 1 (13.9%) and Component 2 (9.2%), with ellipses representing 95% confidence intervals.

Gene expression profiling of the GSE54388 dataset, containing 20,842 gene features, was visualized using a heatmap and revealed two distinct sample clusters (Figure 2A). Notably, most genes showed increased expression in the OVCA group. Additionally, a PLS-DA plot demonstrated a clear separation between normal and OVCA samples, confirming robust clustering by disease status (Figure 2B).

### 3.2. Immune Characteristics Between Normal and OVCA Groups

**Figure 3:**
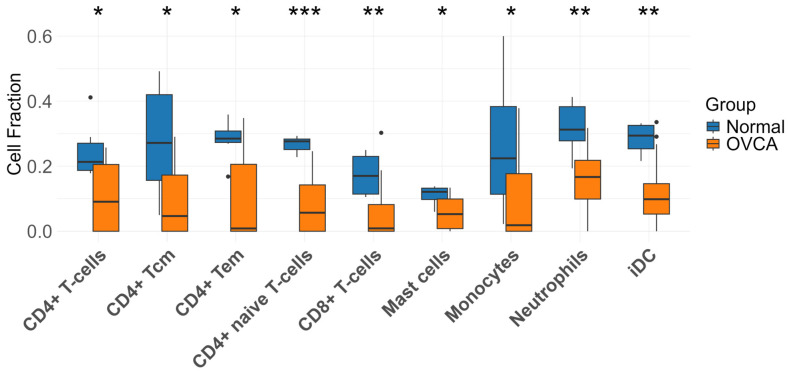
Immune cell infiltration analysis comparing normal and OVCA samples based on the relative abundance of immune cell types. Box plots represent infiltration levels for CD4^+^ T-cells, CD4^+^ central memory T-cells (Tcm), effector memory T-cells (Tem), naïve CD4^+^ T-cells, CD8^+^ T-cells, mast cells, monocytes, neutrophils, and immature dendritic cells (iDC). Orange boxes denote OVCA samples and blue boxes denote normal samples. Y-axis indicates estimated infiltration levels; statistical differences were assessed between groups; * *p* < 0.05, ** *p* < 0.01, *** *p* < 0.001.

To characterize immune cell infiltration, we applied the xCell algorithm to the GSE54388 dataset. OVCA samples exhibited significantly reduced proportions of CD4+ T-cells, CD4+ Tcm, CD4+ Tem, CD4+ naïve T-cells, CD8+ T-cells, mast cells, monocytes, neutrophils, and immature dendritic cells (iDC), relative to normal samples (Figure 3).

### 3.3. Gene Set Enrichment Analysis

**Figure 4:**
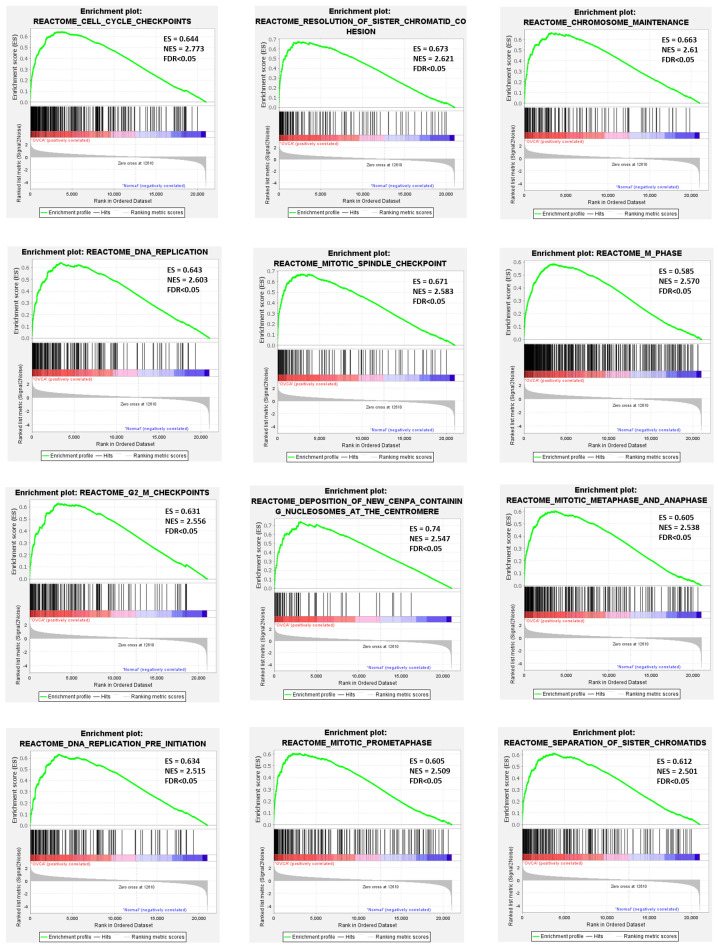
GSEA of Reactome pathways enriched in OVCA. Each enrichment plot illustrates a specific Reactome pathway with the following elements: the green curve represents the enrichment score (ES); tick marks along the x-axis indicate the positions of genes within the ranked list; and the color gradient at the bottom reflects the rank metric score. Normalized enrichment score (NES) and false discovery rate (FDR) are shown for each pathway.

GSEA revealed that several Reactome pathways related to cell cycle were positively enriched in the OVCA group. Specifically, most genes in the tissue samples of OVCA patients were significantly enriched (FDR < 0.05) in cell cycle checkpoints (NES = 2.773), resolution of sister chromatid cohesion (NES = 2.621), chromosome maintenance (NES = 2.61), DNA replication (NES = 2.603), mitotic spindle checkpoint (NES = 2.583), M phase (NES = 2.57), G2-M checkpoints (NES = 2.556), deposition of new CENPA containing nucleosomes at the centromere (NES = 2.547), mitotic metaphase and anaphase (NES = 2.538), DNA replication pre-initiation (NES = 2.515), mitotic prometaphase (NES = 2.509), and separation of sister chromatids (NES = 2.501). These results indicate that pyroptosis-related genes may serve as critical regulators of cell cycle dynamics in ovarian cancer progression (Figure 4).

### 3.4. Identification of Differentially Expressed Genes and Pyroptosis-Related Genes

**Figure 5:**
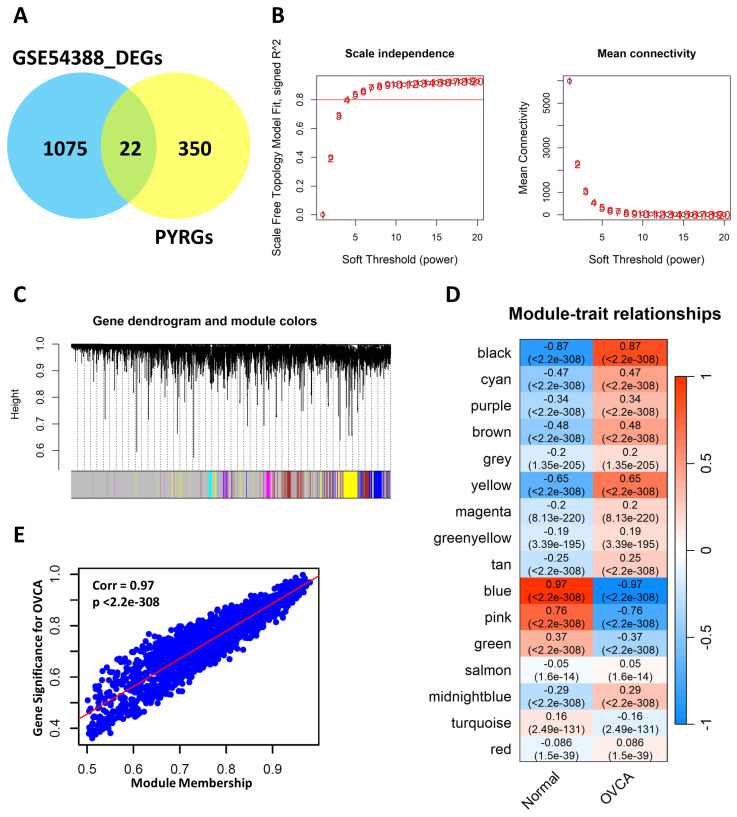
Identification of PYRDEGs and key modules related to OVCA. (**A**) Venn Diagram showing the overlap between DEGs (*n* = 1097) and PYRGs (*n* = 372), identifying 22 intersecting genes (PYRDEGs) selected for further analysis. (**B**) The left plot illustrates the scale-independence index (R^2^ = 0.8) as a function of soft-thresholding power. The right plot displays corresponding mean connectivity, used to determine the optimal soft-thresholding value for WGCNA construction. (**C**) Hierarchical clustering tree of genes grouped into modules using WGCNA. Distinct color blocks beneath the dendrogram represent gene modules identified based on similar expression patterns. (**D**) Module-trait relationship heatmap showing the relationship between gene modules and clinical conditions (Normal vs. OVCA). Each cell includes the correlation coefficient (r) and *p*-value; red indicates positive correlation, blue indicates negative correlation, with color intensity reflecting correlation strength. (**E**) Correlation analysis between gene significance and module membership in the blue module, demonstrating a strong association with clinical traits (r = 0.97; *p* < 2.2 × 10^−308^).

Using GEO2R, we identified 1097 differentially expressed genes (DEGs) in the GSE54388 dataset, including 800 upregulated and 297 downregulated genes. A set of 372 PYRGs was obtained from a previous study, and intersected with the DEGs, yielding 22 pyroptosis-related differentially expressed genes (PYRDEGs): CASP1, CDK1, CEP55, CHMP4C, DNMT1, EZH2, HTRA1, IFI16, IL18, KIF23, LY96, MELK, MKI67, MMP1, MST1, MUC20, NLRP7, PAK2, PKM, PTX3, TNFSF13B, and VDR (Figure 5A and Table 1).

### 3.5. Identification of Key Modules Related to OVCA Using WGCNA

To explore the potential regulatory role of pyroptosis-related genes in OVCA, WGCNA was performed using the GSE54388 microarray dataset, comprising 54,675 gene features across 22 samples. After data cleaning and filtering, 23,520 genes were retained. With a selected soft threshold (Figure 5B), a scale free co-expression network was constructed to identify gene features related to OVCA. A total of 16 gene modules were identified (Figure 5C), of which the blue module containing 1668 genes exhibited the strongest correlation with OVCA (r = −0.97, *p* < 2.2 × 10^−308^) (Figure 5D). As illustrated in Figure 5E, module membership (x-axis) and gene significance (y-axis) revealed strong clustering of genes linked to OVCA. Applying thresholds of MM > 0.6 and GS > 0.5, we extracted 1445 hub genes from the blue module (Table S2).

### 3.6. Identification and Analysis of Hub-Genes in the Blue Module

**Figure 6:**
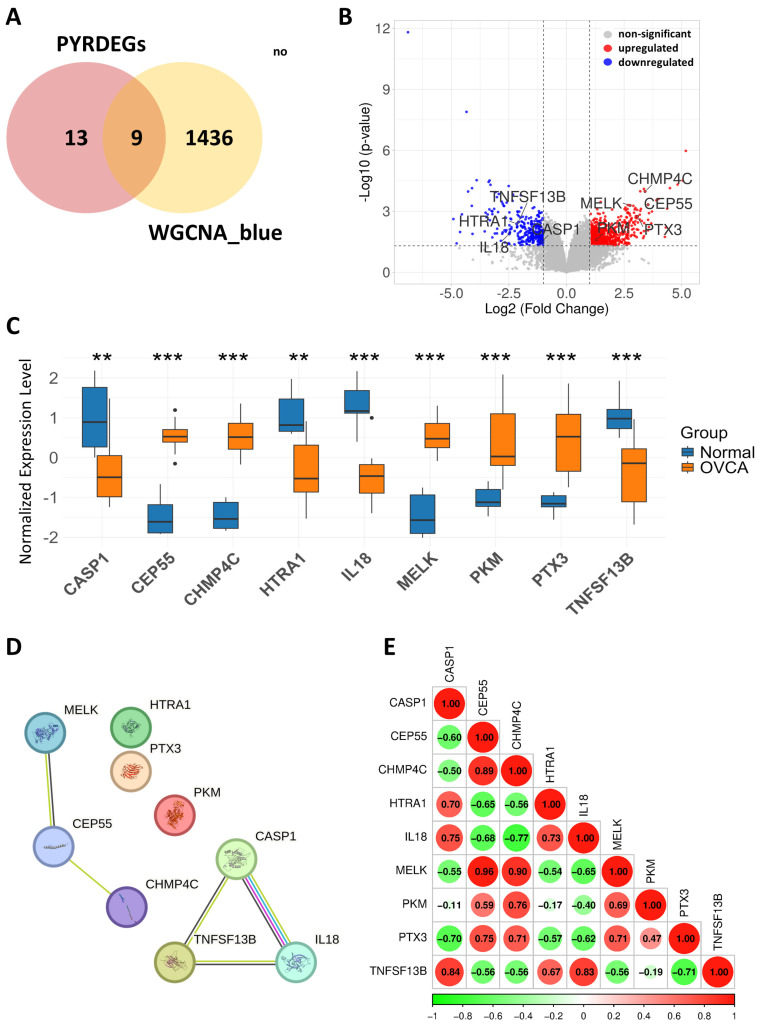
Identification, expression profiling, and network behavior of hub-genes. (**A**) Venn Diagram depicts the intersection between PYRDEGs (*n* = 22) and blue module genes (*n* = 1445), identifying 9 overlapping hub-genes. (**B**) Volcano plot displays 9 hub-genes based on −log_10_ (*p*-value) vs. log_2_ fold change. Red dots represent upregulated genes, blue dots represent downregulated genes, and gray dots denote non-significant genes. Selected hub-genes are labeled. (**C**) Boxplot illustrates normalized expression levels of representative hub-genes across OVCA and normal samples in GSE54388 dataset. Boxes show interquartile ranges; whiskers indicate variability; statistical significance highlights gene expression differences between groups. (**D**) PPI network of 9 hub-genes constructed using STRING, revealed two significant clusters with strong connectivity. Edge colors indicate the type of supporting evidence: cyan lines represent curated database interactions, pink lines represent experimentally determined interactions, yellow lines represent associations identified through textmining, and black lines represent co-expression evidence. (**E**) Correlation heatmap represents Pearson correlations among hub-genes. Red circles indicate positive correlations, green circles indicate negative correlations, and circle size reflects correlation strength. ** *p* < 0.01, *** *p* < 0.001.

Intersection analysis between the 1445 blue module genes and 22 previously identified PYRDEGs yielded nine overlapping hub genes: CASP1, CEP55, CHMP4C, HTRA1, IL18, MELK, PKM, PTX3, and TNFSF13B (Figure 6A). A volcano plot highlighted these nine genes among all deregulated genes (Figure 6B). Wilcoxon rank-sum testing indicated that all nine hub genes were significantly differentially expressed between normal and OVCA groups (*p* < 0.01). Specifically, five genes (CEP55, CHMP4C, MELK, PKM, and PTX3) were upregulated in OVCA, whereas four (CASP1, HTRA1, IL18, and TNFSF13B) were downregulated (Figure 6C). The protein–protein interaction network revealed a strong interaction cluster among CASP1, IL18, and TNFSF13B, indicating their coordinated roles in immune and inflammatory pathways. In contrast, a separate module comprising CEP55, MELK, and CHMP4C was associated with cell cycle regulation, while PKM, PTX3, and HTRA1 showed limited connectivity, suggesting roles in independent biological processes (Figure 6D). Correlation analysis further demonstrated strong positive associations between CEP55 and MELK (r = 0.96), CHMP4C (r = 0.89), PTX3 (r = 0.75), and PKM (r = 0.59). Negative correlations were observed between CEP55 and IL18 (r = −0.68), HTRA1 (r = −0.65), CASP1 (r = −0.60), and TNFSF13B (r = −0.56) (Figure 6E).

### 3.7. Functional Enrichment Analysis of Hub-Genes

**Figure 7:**
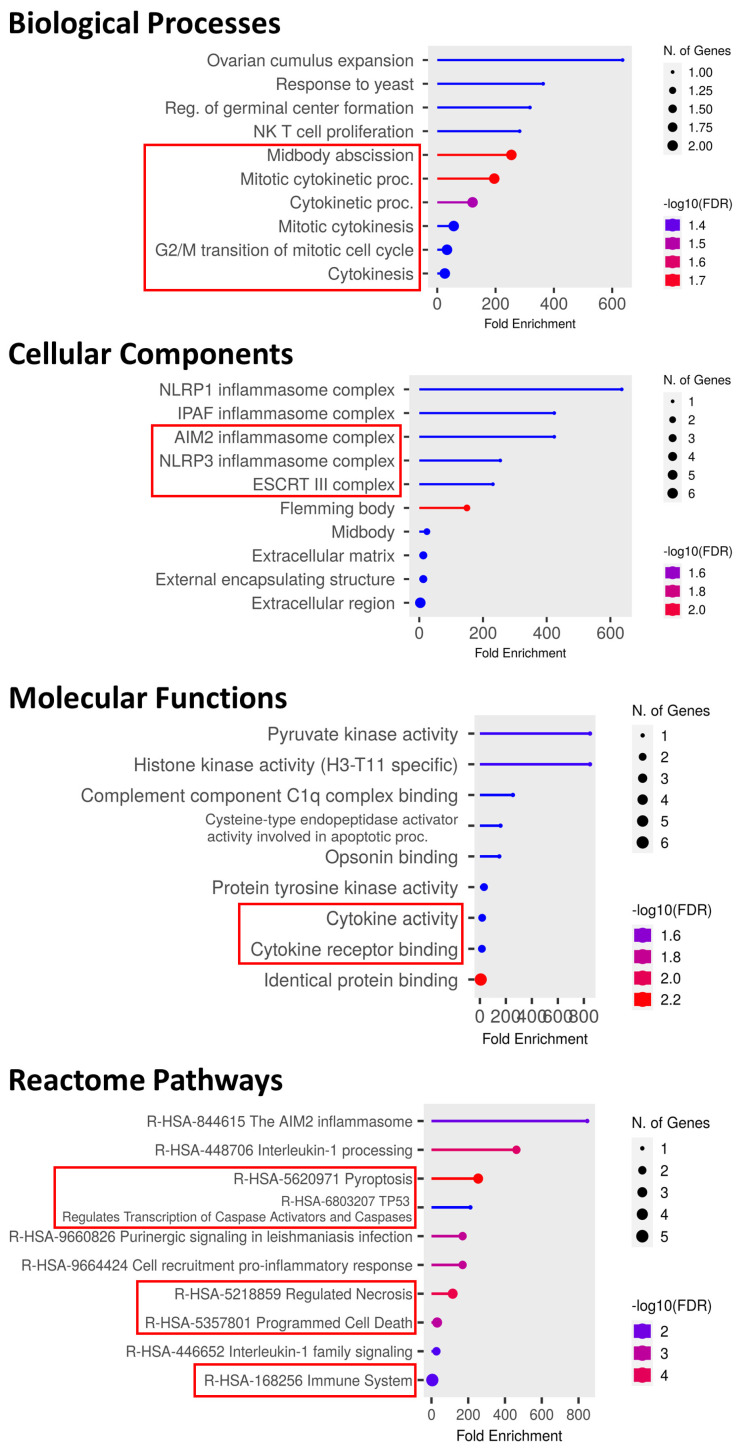
Functional enrichment analysis of hub genes using GO and Reactome pathways. The lollipop plot illustrates significantly enriched biological processes, cellular components, molecular functions, Reactome pathways, with fold enrichment on the x-axis, dot size indicating gene count, and color representing statistical significance (−log_10_ FDR).

Gene Ontology (GO) and Reactome pathway analyses were performed to characterize the important biological functions of the identified hub genes. GO biological process (BP) terms included enrichment in midbody abscission, mitotic cytokinetic processes, cytokinetic processes, mitotic cytokinesis, G2/M transition of mitotic cell cycle, and cytokinesis. Cellular component (CC) analysis showed AIM2/NLRP3/ESCRT-III inflammasome complexes. Molecular function (MF) analysis revealed involvement in cytokine activity and cytokine receptor binding. Reactome analysis indicated significant enrichment in pathways related to pyroptosis, TP53 regulates transcription of caspase activators and caspases, regulated necrosis, programmed cell death, and immune system (Figure 7).

### 3.8. Identification of Core Pyroptosis-Related Hub-Genes Using Machine Learning

**Figure 8:**
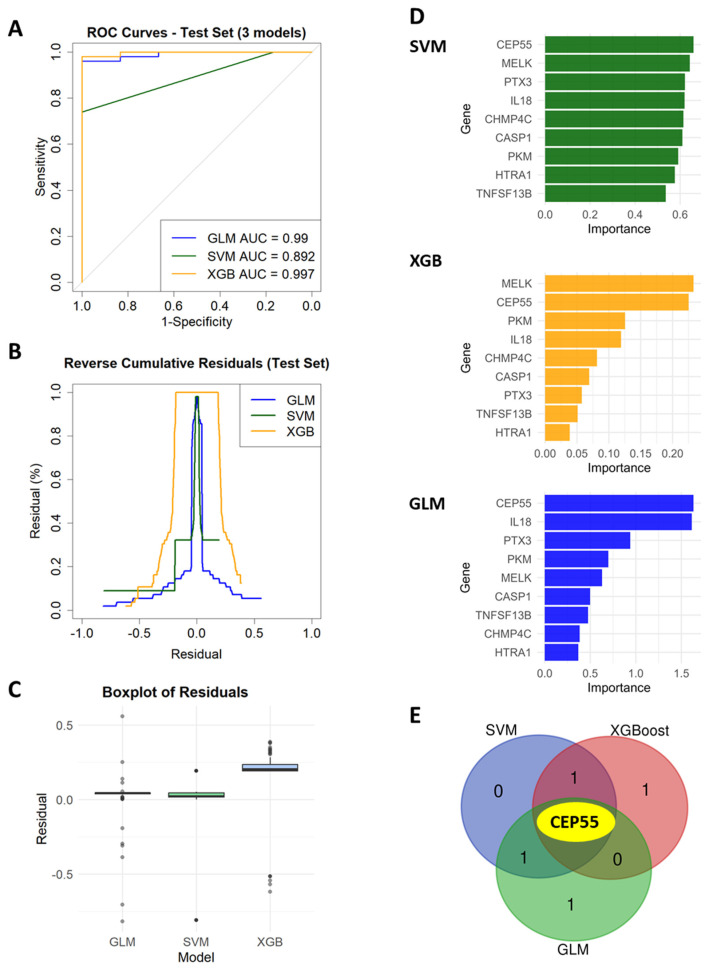
Model performance evaluation and feature selection across multiple machine learning algorithms for classification of OVCA samples. (**A**) ROC curves of SVM (darkgreen), XGBoost (orange), and GLM (blue) algorithms, illustrating diagnostic performance. (**B**,**C**) Residual distribution graph and Residual boxplot supporting model stability. (**D**) Bar plots displaying the top-ranking genes identified as important features by SVM, XGBoost, and GLM. (**E**) Venn diagram illustrating shared and unique important genes among the three algorithms. The central overlap identifies CEP55 as a common feature across all algorithms. (XGB = XGBoost).

All three machine learning algorithms: SVM, XGBoost, and GLM demonstrated strong discriminative ability in the independent test set, with AUC values of 0.997 for XGBoost, 0.892 for SVM, and 0.99 for GLM (Figure 8A). ROC curve analysis confirmed high diagnostic performance, while residual analyses, including boxplots and reverse cumulative distribution plots, supported model stability (Figure 8B,C). Feature importance rankings consistently highlighted CEP55 as the top predictive gene, alongside MELK, PKM, IL18, and PTX3 depending on the algorithm (Figure 8D). By intersecting the top 3 ranked features across all three classifiers, CEP55 emerged as the shared pyroptosis-related core hub gene, highlighting as potential biomarker candidate under stringent validation and regularization conditions (Figure 8E).

### 3.9. Diagnostic Performance, Validation, and Immune Cell Correlation of CEP55

**Figure 9:**
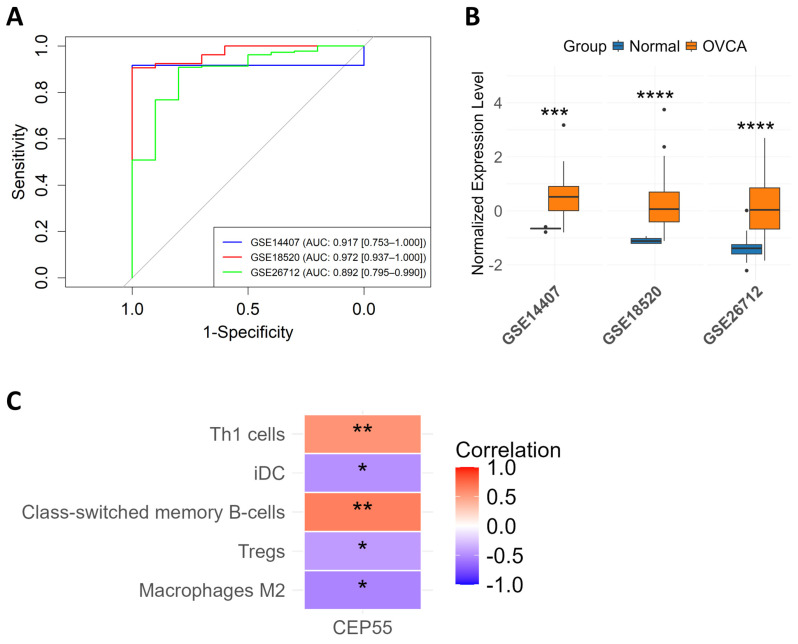
Diagnostic accuracy, validation and immune cell correlation of CEP55. (**A**) ROC curves assessing the diagnostic value of CEP55 across validation datasets GSE14407, GSE18520, and GSE26712 reveal strong predictive performance, with AUC scores of 0.917, 0.972, and 0.892, respectively. (**B**) Box plots illustrating CEP55 expression across OVCA and normal tissues in three validation datasets reveal consistently elevated levels in OVCA samples, with median values, interquartile ranges, and whiskers displayed. (**C**) A heatmap visualizing the correlation between immune cell populations and CEP55 expression in OVCA versus normal samples highlights distinct patterns among Th1 cells, iDCs, class-switched memory B cells, Tregs, and M2 macrophages, with blue-to-red color gradients indicating negative to positive correlations. (* *p* < 0.05, ** *p* < 0.01, *** *p* < 0.001, **** *p* < 0.0001).

To assess the diagnostic performance of CEP55, ROC curve analysis was performed across three independent datasets. The AUC values for CEP55 were 0.917 in GSE14407, 0.972 in GSE18520, and 0.892 in GSE26712. The ROC analysis demonstrated maximum sensitivity and specificity values of 91.7% and 100%, respectively, confirming the high diagnostic power of CEP55 and validating the effectiveness of machine learning in identifying core biomarker genes (Figure 9A). Expression validation across the three datasets revealed significant upregulation of CEP55 in OVCA samples compared to normal ovarian tissues, supporting its potential as a reliable diagnostic biomarker for ovarian cancer (Figure 9B). Correlation analysis showed that CEP55 expression was positively associated with Th1 cells (r = 0.55, *p* < 0.01) and class-switched memory B cells (r = 0.65, *p* < 0.01), while negatively correlated with immature dendritic cells (r = −0.48, *p* < 0.05), regulatory T cells (Tregs) (r = −0.43, *p* < 0.05), and M2 macrophages (r = −0.53, *p* < 0.05) (Figure 9C).

### 3.10. Construction of mRNA-miRNA, mRNA-RBP, and mRNA-TF Interaction Networks

**Figure 10:**
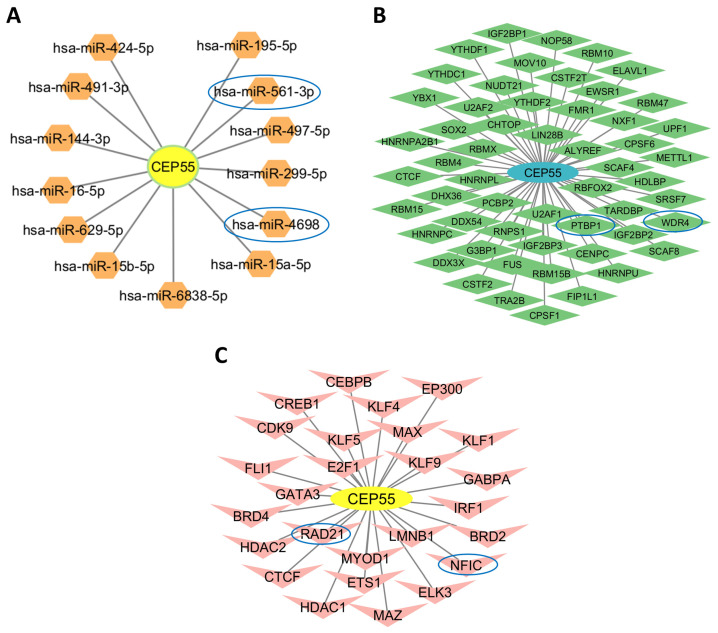
Predicted regulatory networks targeting CEP55. (**A**) The CEP55-miRNA interaction network shows predicted microRNAs that may regulate CEP55 post-transcriptionally in OVCA, with hsa-miR-561-3p and hsa-miR-4698 identified as the most influential nodes. (**B**) The CEP55-RBP network displays predicted RBPs that interact with CEP55 transcripts, potentially affecting mRNA stability and translation, with PTBP1 and WDR4 emerging as the most influential regulators. (**C**) The CEP55-TF regulatory network highlights TFs predicted to act upstream of CEP55, suggesting possible transcriptional control pathways, with RAD21 and NFIC identified as the most influential nodes. The top 2 influential nodes interacting with CEP55 are circled in blue.

To predict miRNAs interacting with the core gene CEP55, data from the miRDB database was utilized, and an mRNA-miRNA interaction network was constructed for visualization. This network included CEP55 and 13 associated miRNA molecules (Figure 10A). RNA-binding proteins (RBPs) interacting with CEP55 were identified using the ENCORI database, and the corresponding mRNA-RBP interaction network was generated, comprising CEP55 and 55 RBPs (Figure 10B). To identify transcription factors (TFs) binding to CEP55, both the CHIPBase and the hTFtarget databases were used. The intersection of TFs predicted from both sources resulted in 26 shared TFs, and the interaction network linking CEP55 with these transcription factors was visualized (Figure 10C).

### 3.11. scRNA-Seq Data Processing and Analysis

**Figure 11:**
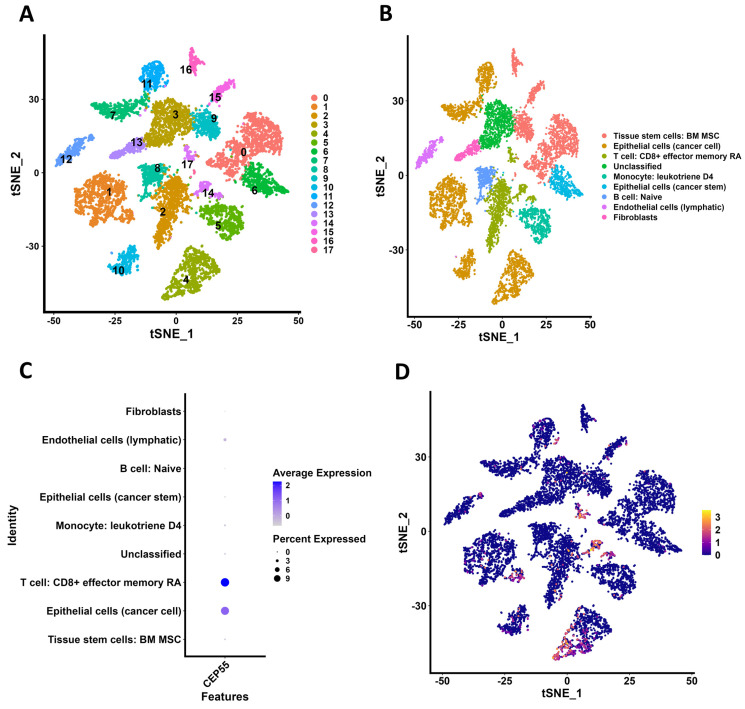
scRNA-seq data processing and expression analysis. (**A**) The t-SNE algorithm divided the cells into clusters using 18 PCs. (**B**) 18 cell clusters were annotated into different cell types. (**C**) Bubble plot of the CEP55 expression level in 18 cell clusters. (**D**) tSNE map of the expression of CEP55 in 18 cell clusters.

Six OVCA samples containing 9885 cells were subjected to further analysis. No correlation was observed between the percentage of mitochondrial genes and sequencing depth (Figure S1A). In contrast, a strong positive correlation (r = 0.89) was found between the number of genes and sequencing depth (Figure S1B). RNA count distributions across the six samples showed wide variability, confirming adequate sequencing depth (Figure S1C), while gene count distributions revealed consistent capture of expressed genes, supporting dataset quality (Figure S1D). The top 10 highly variable genes, including immunoglobulin family members, are shown in a volcano plot (Figure S1E). PCA based on these variable genes demonstrated clear clustering of the six samples, indicating distinct transcriptomic profiles (Figure S1F). Dimheatmap visualization identified gene expression drivers across principal components (Figure S2), and JackStraw analysis confirmed the statistical significance of PCs 1–20 (Figure S1G). The Elbow-Plot further indicated that the first 18 components captured the majority of biologically relevant variance (Figure S1H). Because no clear elbow point was observed and all *p*-values were <0.01, cumulative percentages were calculated for each PC; Figure S1I showed that 18 was the final point where the variation change exceeded 0.1%, so 18 was selected as the PC value. The t-SNE plot identified 18 distinct clusters, reflecting heterogeneous cell populations (Figure 11A). A total of 4777 genes were used as markers for these clusters, and the top 10 significantly differentially expressed marker genes per cluster are illustrated in a heatmap (Figure S3). Cell type annotation mapped clusters to specific populations, including cancer epithelial cells and TEMRA cells. (Figure 11B). CEP55 expression was enriched in both cancer epithelial cells and TEMRA cells (Figure 11C) with higher levels in clusters 2, 14, and 17 (TEMRA cells) and clusters 1, 4, 7, 10, and 11 (cancer epithelial cells) (Figure 11D). These results indicate that CEP55 is predominantly expressed in tumor-associated epithelial cells in ovarian cancer tissue, and we hypothesize that CEP55 influences both TEMRA and epithelial cancer cells to regulate tumor cell biology.

### 3.12. Gene Expression and Survival Analysis

**Figure 12:**
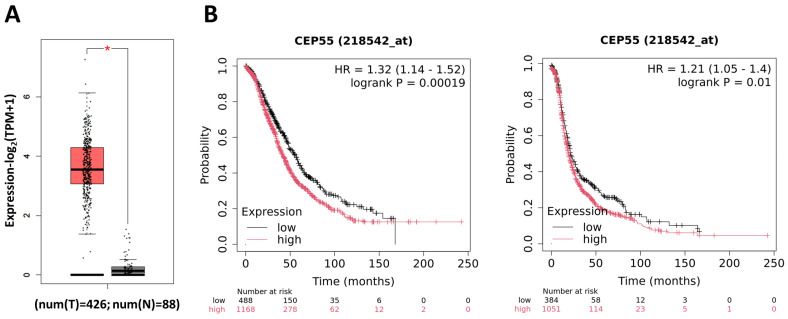
Analysis of the link between CEP55 expression in ovarian cancer through (**A**) GEPIA2 database (T = ovarian cancer; gray = normal ovarian tissues) and (**B**) overall survival (OS) or progression-free survival (PFS) using the KM-plotter database (* *p* < 0.05).

Analysis using GEPIA2 and Kaplan–Meier Plotter databases revealed that CEP55 is highly expressed in ovarian cancer (Figure 12A) and shows a negative correlation with overall survival (OS) and progression-free survival (PFS) in ovarian cancer (Figure 12B).

## 4. Discussion

Ovarian cancer remains a major clinical challenge due to late diagnosis, recurrence, and therapy resistance. These factors underscore the urgent need for novel molecular biomarkers and therapeutic strategies to better understand OVCA pathogenesis and progression [43]. In this study, we applied transcriptomic and network-based bioinformatics approaches to identify pyroptosis-related hub genes and their regulatory networks in high-grade serous ovarian carcinoma (HGSOC).

Pyroptosis, an inflammatory form of programmed cell death, has emerged as a paradoxical mechanism in cancer. While it can mediate tumor suppression by eliminating damaged cells, its associated release of pro-inflammatory cytokines may also enhance tumorigenesis by fostering a pro-tumor microenvironment [44]. As such, targeting pyroptosis-related genes presents a promising therapeutic strategy for modulating cancer progression [45]. Our transcriptomics analysis revealed distinct clustering between OVCA and normal samples, supported by heatmap and PLS-DA visualization. Immune cell infiltration analysis demonstrated significant reductions in CD4^+^ T cells (naïve, Tcm, Tem), CD8^+^ T cells, mast cells, monocytes, neutrophils, and immature dendritic cells (iDCs). These findings suggest that OVCA suppresses critical immune populations involved in antigen presentation, cytotoxic responses, and tumor surveillance, thereby facilitating immune evasion, consistent with prior observations [46].

GSEA demonstrated activation of cell cycle pathways, including DNA replication, mitotic checkpoints, centromere maintenance, and nucleosome deposition at the centromere (NES > 2.5, FDR < 0.05). These results support earlier findings that cell cycle dysregulation contributes to genomic instability and malignant progression in HGSOC. Multiple modes of cell cycle arrest likely reflect both intertumoral heterogeneity and checkpoint malfunction [47]. A separate study reported high CEP55 expression associated with tumor proliferation and poor prognosis [48]. However, that study was limited to a small cohort and did not assess immune infiltration, which our analysis identifies as a critical factor in CEP55’s dual role.

Our analysis of pyroptosis-related genes revealed that 22 were deregulated in OVCA, and network analysis via WGCNA identified 1445 genes within the blue module highly correlated with disease state. Intersection analysis identified nine hub genes: CASP1, CEP55, CHMP4C, HTRA1, IL18, MELK, PKM, PTX3, and TNFSF13B. Prior studies have shown that overexpression of CEP55 in ovarian cancer destabilizes chromosome segregation by over-stabilizing spindle microtubules, contributing to chromosomal instability [49]. Additionally, CEP55 overexpression correlates with advanced disease stage, metastasis, and recurrence, while its knockdown suppresses invasion and migration in epithelial ovarian carcinoma [50]. Although downregulation of HTRA1 has been linked to improved outcomes [51], these findings were based on bulk transcriptomic data; our single-cell analysis suggests that cell-type specificity may alter its prognostic relevance. Prior reports of caspase-1 and IL-18 upregulation [52] emphasized inflammatory signaling, but they did not explore how these cytokines interact with cell cycle dysregulation, a connection highlighted in our study.

Functional enrichment revealed that hub genes were significantly involved in cytokinesis, mitotic processes, and midbody abscission, critical for cell division. CEP55 in particular localizes to the midbody and facilitates abscission via ESCRT-III recruitment [53]. Inflammatory mediators such as NLRP3 and AIM2 were highlighted as central regulators of inflammasome-driven pyroptosis, confirming their dual role in tumor progression and immune modulation [52,54]. ESCRT-III role in membrane repair during pyroptosis and necroptosis further implicates it in tumor cell survival and immune evasion [55]. Collectively, these findings indicate that dysregulation across cell division, immune response, membrane remodeling, and metabolic pathways converges to promote disease progression and alter immune dynamics.

Using three machine learning algorithms (SVM, XGBoost, and GLM), CEP55 was identified as a core pyroptosis-related hub gene. Notably, CEP55 has been reported as overexpressed across multiple human cancers, including gastric, hepatocellular (HCC), lung, nasopharyngeal, oral cavity squamous cell carcinoma (OCSCC), urinary bladder, renal, breast, and colorectal cancers [56,57,58,59,60,61,62,63,64], supporting its potential biological relevance in OVCA.

A recent study identified CEP55 as a clinically relevant biomarker in ovarian cancer, with its expression levels significantly associated with patient survival and tumor progression [65]. Correlation analysis of CEP55 with immune cell types revealed both significant positive and negative relationships. Notably, an imbalance in the Th1/Th2 ratio, especially a shift toward Th2 dominance has been associated with impaired immune surveillance and increased tumor progression [66,67]. Xie et al. reported a positive correlation between CEP55 expression and Th2 cell infiltration, suggesting that CEP55 may contribute to this immunological imbalance [68]. In contrast, our study revealed a positive correlation between CEP55 and Th1 cells, suggesting a potential shift toward Th1-mediated immunity. This finding may indicate a dual role for CEP55 depending on tumor stage, supporting an anti-tumor immune response and potentially reflecting a more favorable prognosis. In addition, CEP55 exhibited a negative correlation with immature dendritic cells (DCs), which play a critical role in initiating adaptive immune responses; reduced levels of DCs may hinder antigen presentation and compromise immune activation [69]. Within the context of ovarian cancer, CEP55 appears to function as a pan-cancer biomarker, associated with both tumor progression and immune cell infiltration. Its elevated expression in this study negatively correlated with regulatory T cells (Tregs), implying a role in promoting an immunosuppressive tumor microenvironment and potentially influencing responses to immunotherapy [70]. Further evidence from breast cancer research demonstrates that SPI1 transcriptionally upregulates CEP55, thereby promoting malignant progression and inducing M2 macrophage polarization, another mechanism contributing to immune suppression [71].

In this study, CEP55 displayed significantly elevated expression across all three independent validation cohorts and was consistently selected as a key gene using three machine learning algorithms. It demonstrated strong diagnostic performance, with area under the curve (AUC) values of 0.917 in GSE14407, 0.972 in GSE18520, and 0.892 in GSE26712. Within the GSE14407 dataset, CEP55 achieved a maximum sensitivity of 91.7% and specificity of 100%, further supporting its diagnostic utility in OVCA. Moreover, CEP55 was found to interact with multiple miRNAs, RBPs, and TFs, highlighting its involvement in post-transcriptional regulation, stability, and gene expression control. It was observed that hsa-miR-561-5p was downregulated in ovarian cancer samples in comparison to the control group [72]. The overexpression of Polypyrimidine Tract Binding Protein 1 (PTBP1) in ovarian cancer cells could promote cell metastasis and colony formation due to the fact that PTBP1 could promote the exon 6B skipping of CDC42 [73]. Double-strand-break repair protein RAD21 was markedly upregulated in ovarian cancer samples, and its high expression was linked to poor differentiation and unfavorable prognosis in patients [74]. Nucleus Factor IC (NFIC) inhibits the progression of epithelial ovarian cancer by regulating the balance of PTEN/TGFβ1/EGR1/BRD4 and SP1/EZH2 to suppress the TBX2/MMPs signaling pathway [75]. CEP55 overexpression synergizes with other oncogenic factors while counteracting tumor-suppressive regulators such as NFIC, making it a critical biomarker and therapeutic target in ovarian cancer.

Evidence from other malignancies also supports CEP55’s oncogenic role. For instance, CEP55 is significantly overexpressed in oral squamous cell carcinoma compared to normal tissues, and its elevated levels are associated with poor survival, cell cycle dysregulation, and reduced immune infiltration, making it both a prognostic biomarker and a potential therapeutic target [76]. Our analyses showed that CEP55 is primarily expressed in cancerous epithelial cells and in TEMRA cells, as seen in single-cell t-SNE and dot plots, suggesting roles in tumor growth and immune modulation. Bulk transcriptomic data confirmed higher CEP55 levels in tumor tissues compared to normal tissues, and survival analyses consistently demonstrated worse outcomes in patients with elevated CEP55 (HR > 1.2, *p* < 0.05). These findings indicate that elevated CEP55 is both a marker of aggressive tumor biology and poor patient survival, while its presence in TEMRA cells suggests that CEP55 may influence immune responses, potentially driving tumor progression and therapy resistance. Collectively, this evidence supports the conclusion that CEP55 impacts ovarian cancer prognosis through its roles in tumor-associated TEMRA cells and epithelial cancer cells.

Despite these promising findings, the study has several limitations. The analyses were entirely based on bioinformatics methods without experimental validation, and the datasets used involved relatively small sample sizes. Future research is needed to validate CEP55’s role and diagnostic accuracy in vivo and to explore its potential as a therapeutic target through functional and mechanistic studies.

## 5. Conclusions

In summary, this study identified nine hub genes (CASP1, CEP55, CHMP4C, HTRA1, IL18, MELK, PKM, PTX3, and TNFSF13B) as potential biomarkers for the diagnosis of OVCA. Among these, CEP55 was notably upregulated in patient samples and emerged primarily as a key regulator of the cell cycle, underscoring its relevance to OVCA pathogenesis. While CEP55 was highlighted through enrichment and machine learning prioritization, its role as a pyroptosis-related biomarker remains to be clarified and warrants further mechanistic investigation. Moreover, CEP55 was shown to participate in a complex regulatory network involving miRNAs, RBPs, and TFs, suggesting multifaceted contributions to disease progression. Validation across bulk and single-cell RNA sequencing datasets confirmed its elevated expression in tumor-associated epithelial cells and TEMRA cells, linking CEP55 to altered immune infiltration and poor survival outcomes. Collectively, these findings provide a framework for advancing early diagnostic tools and targeted therapeutic strategies, while emphasizing the need for deeper mechanistic studies to establish CEP55 precise role in Ovarian cancer biology.

## Figures and Tables

**Table 1:** List of 22 Pyroptosis-Related Differentially Expressed Genes (PYRDEGs).

Gene Symbol	Gene Title	−log10 (*p*-Value)	Log2 (Fold Change)	UP/DOWN
CASP1	caspase 1	1.49	−1.09	DOWN
CDK1	cyclin dependent kinase 1	2.02	3.36	UP
CEP55	centrosomal protein 55	3.32	3.54	UP
CHMP4C	charged multivesicular body protein 4C	3.97	3.41	UP
DNMT1	DNA (cytosine-5-)-methyltransferase 1	1.33	1.32	UP
EZH2	enhancer of zeste 2 polycomb repressive complex 2 subunit	1.44	2.03	UP
HTRA1	HtrA serine peptidase 1	2.38	−2.16	DOWN
IFI16	interferon gamma inducible protein 16	1.51	−1.65	DOWN
IL18	interleukin 18	1.88	−2.49	DOWN
KIF23	kinesin family member 23	1.58	2.5	UP
LY96	lymphocyte antigen 96	1.75	−1.49	DOWN
MELK	maternal embryonic leucine zipper kinase	3.29	2.76	UP
MKI67	marker of proliferation Ki-67	1.49	1.54	UP
MMP1	matrix metallopeptidase 1	2.17	1.7	UP
MST1	macrophage stimulating 1	1.64	−1.55	DOWN
MUC20	mucin 20, cell surface associated	1.53	1.6	UP
NLRP7	NLR family pyrin domain containing 7	1.70	1.15	UP
PAK2	p21 (RAC1) activated kinase 2	1.43	1.93	UP
PKM	pyruvate kinase, muscle	1.60	1.3	UP
PTX3	pentraxin 3	2.75	3.08	UP
TNFSF13B	tumor necrosis factor superfamily member 13b	2.47	−2.08	DOWN
VDR	vitamin D (1,25-dihydroxyvitamin D3) receptor	1.93	1.26	UP

## Data Availability

The data presented in this study using GSE54388, GSE14407, GSE18520, GSE26712, and GSE147082 datasets are publicly available in the NCBI Gene Expression Omnibus database (GEO, http://www.ncbi.nlm.nih.gov/geo, accessed on 25 March 2025).

## Supplementary Materials

The following supporting information can be downloaded at: https://www.mdpi.com/article/10.3390/genes17050595/s1, Figure S1: scRNA-seq data processing and analysis; Figure S2: Dimheatmap used to visualize the top genes contributing to principal components (PCs) in PCA; Figure S3: Heatmap showing top 10 significantly differentially expressed marker genes of each cluster; Table S1: List of pyroptosis-related genes used in this study (*n* = 372); Table S2: List of identified genes in the blue module (*n* = 1445; MM > 0.6, GS > 0.5).

## Abbreviations

AUC	Area Under the Curve
CASP1	Caspase 1
CEP55	Centrosomal Protein 55
DEG	Differentially Expressed Gene
GEPIA2	Gene Expression Profiling Interactive Analysis2
GO	Gene Ontology
GLM	Generalized Linear Model
GSEA	Gene Set Enrichment Analysis
HGSOC	High-Grade Serous Ovarian Cancer
KMplot	Kaplan–Meier Plot
MM	Module Membership
OVCA	Ovarian Cancer
PPI	Protein–Protein Interaction
PYRG	Pyroptosis-Related Gene
scRNA-seq	single-cell RNA sequencing
SVM	Support Vector Machine
TEMRA	T cell: CD8+ effector memory RA
WGCNA	Weighted Gene Co-expression Network Analysis
XGBoost	eXtreme Gradient Boosting
