# Supplementary material for: Pyroptosis-Related Gene Signatures and Immune Modulation in Ovarian Cancer: Insights from Multi-Omics and Machine Learning
Source: Genes (Basel). 2026 May 21;17(5):595. doi: 10.3390/genes17050595 (PMC13206606; doi:10.3390/genes17050595)
Supplement: Supplementary file 1 [file genes-17-00595-s001.zip › Supplementary Figures.pdf]

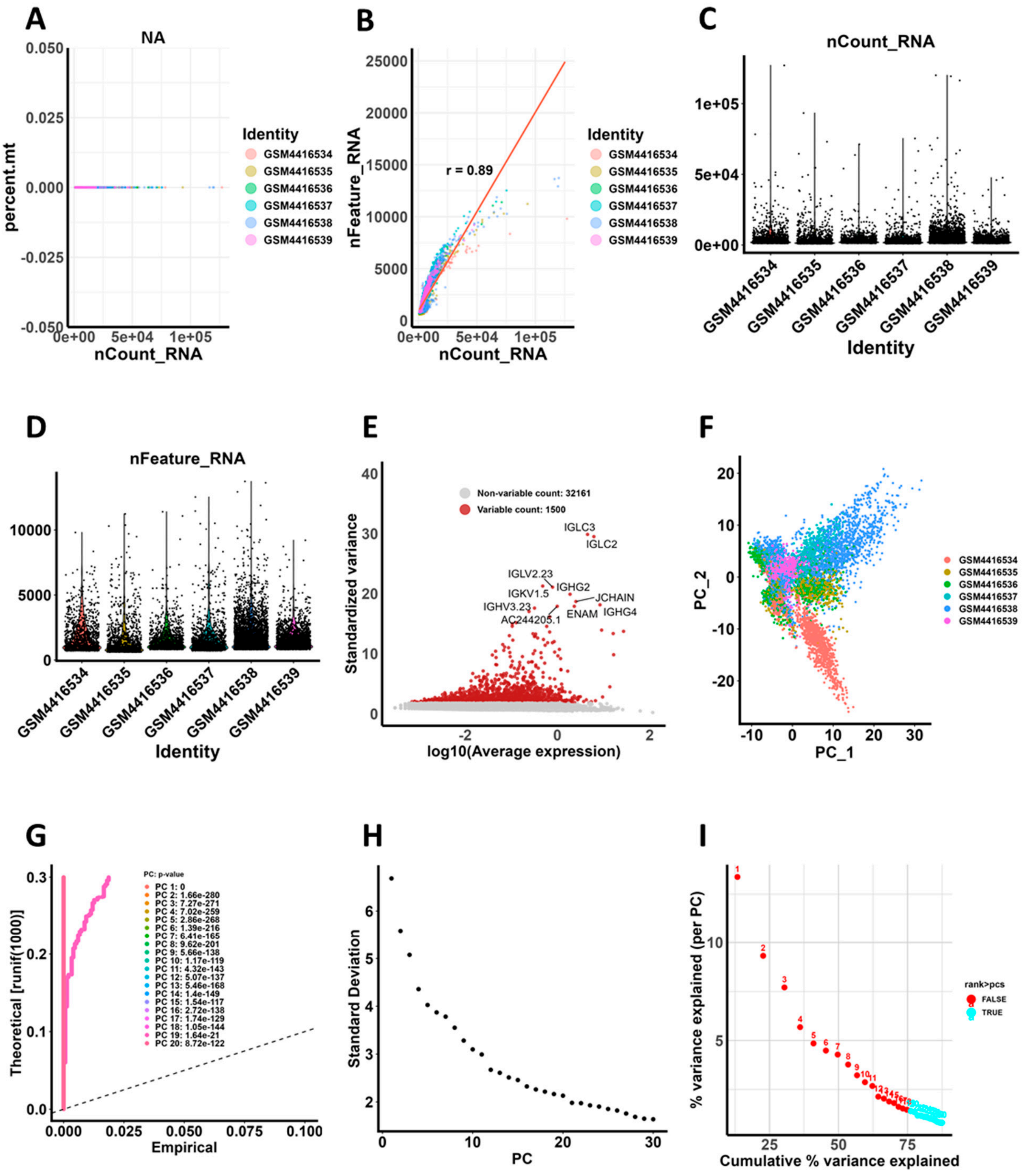

Figure S1. scRNA-seq data processing and analysis. (A) The correlation analysis between sequencing depth and mitochondrial genes (NA= no correlation). B The correlation analysis between the sequencing depth and number of genes using Pearson's method (r=0.89). (C) The sequencing depth of 9885 cells from 6 ovarian cancer patients. (D) The number of genes of 9885 cells from 6 ovarian cancer patients. (E) Detection of the highly variable genes across the cells in

volcano plot, the top 10 genes were marked out. (F) PCA plot of scRNA-seq samples from 6 patients. (G) The p values of PCs from 1-20 calculated by JackStraw function. (H) The standard deviation of 1-30 PCs calculated using ElbowPlot function. (I) Calculation of the cumulative percentages for each PC, 18 is the last point where change of % of variation is more than 0.1%.

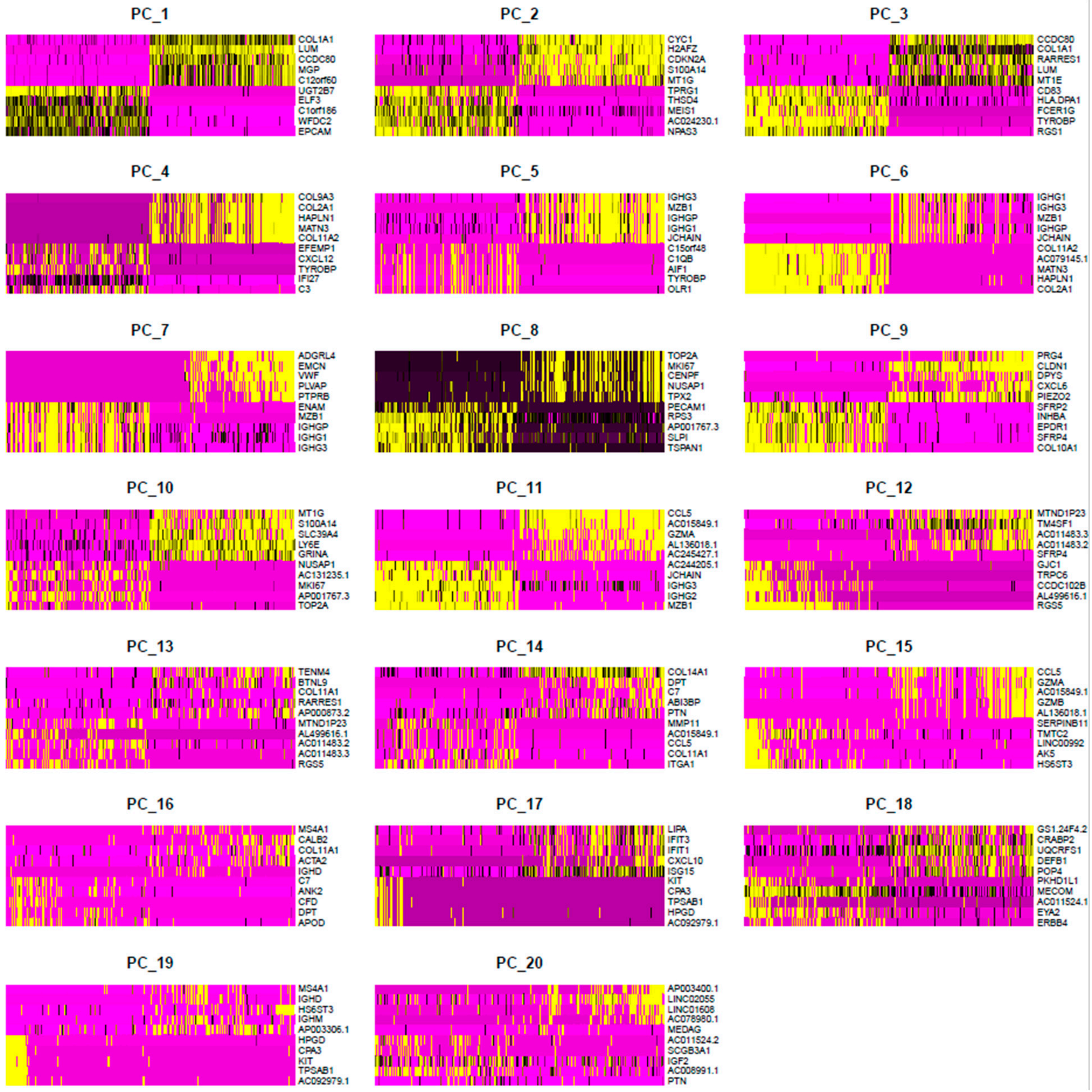

Figure S2. Dimheatmap used to visualize the top genes contributing to principal components (PCs) in PCA.

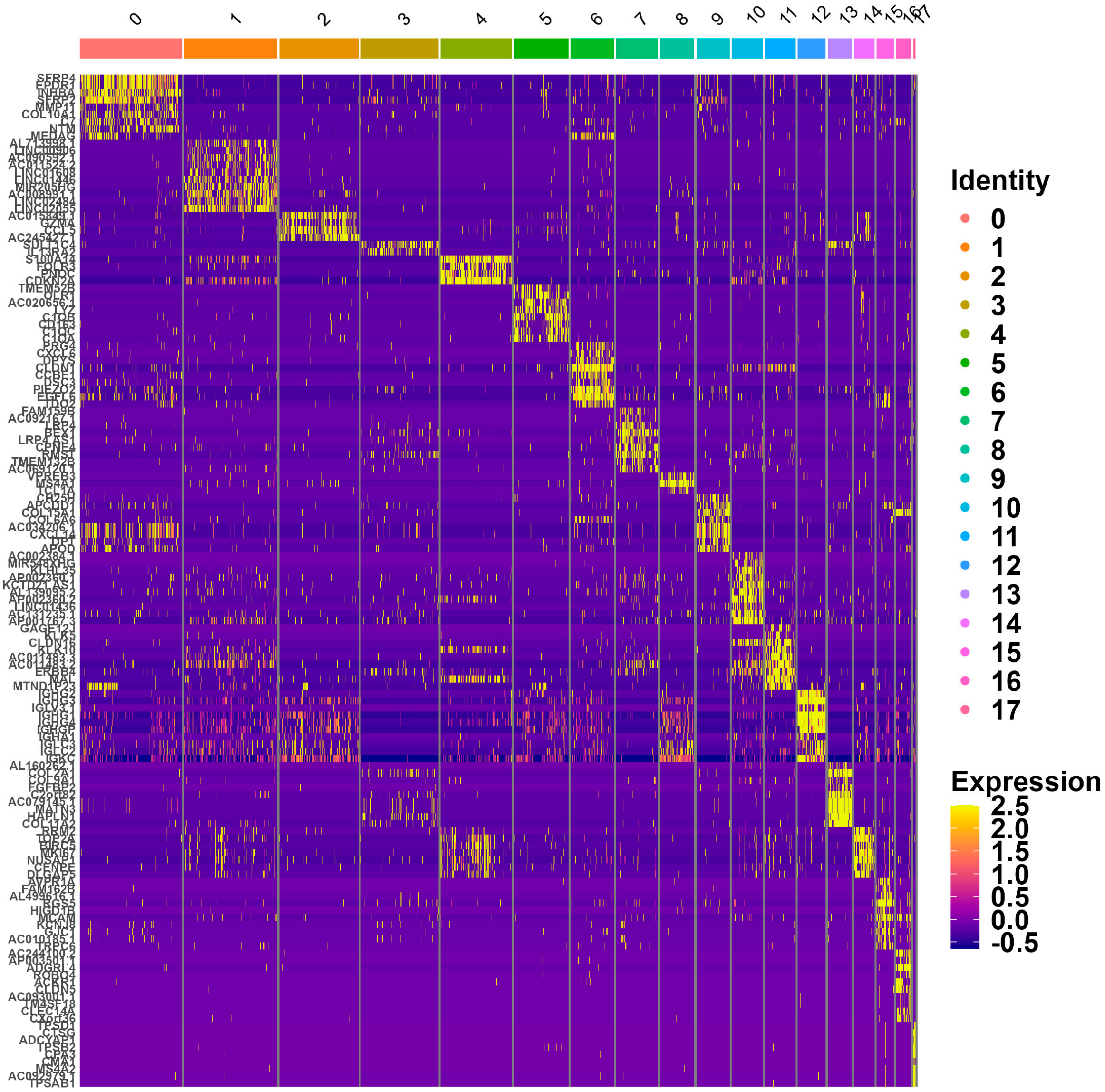

Figure S3. Heatmap showing top 10 significantly differentially expressed marker genes of each cluster.
